# Discussing sexuality with patients with neurological diseases: A survey among neurologists working in Saudi Arabia

**DOI:** 10.3389/fneur.2023.1083864

**Published:** 2023-01-31

**Authors:** Daifallah Mohammed Almalki, Mamdouh Ali Kotb, Anas Mohammed Albarrak

**Affiliations:** ^1^Department of Internal Medicine, College of Medicine, Prince Sattam bin Abdulaziz University, Al-Kharj, Saudi Arabia; ^2^Neurology Department, Faculty of Medicine, Minia University, Minia, Egypt

**Keywords:** sexuality, neurologic disease, stroke, multiple sclerosis, Parkinson's disease

## Abstract

**Background:**

Neurological diseases frequently affect sexual activity, and the resulting sexual dysfunction can cause much distress for patients. However, despite the importance of such complaints, neurologists frequently do not ask patients about their sexual symptoms or how their neurological illness and medications are affecting their sexual health. This study aimed to identify these difficulties as well as potential obstructions to conversations for addressing sexual dysfunction in patients with neurological diseases.

**Methods:**

This cross-sectional study was performed by sending invitation letters and questionnaires to registered neurologists in Saudi Arabia. The questionnaire was constructed to determine the possibility of discussing sexual activities and function with patients with neurological diseases and the possible obstacles neurologists face in this regard. Statistical analyses were performed using the Statistical Package of Social Sciences (SPSS) program version 25, and *p*-values of <0.05 were considered statistically significant.

**Results:**

A total of 258 of 750 neurologists (34.4%) returned the survey, of which 252 had completed the entire survey; therefore, their responses were considered suitable for further analysis. The majority of the respondents (63.1%) seldom discussed sexuality with their patients, more than half of the participants never discussed sexuality with female patients, and patients aged 60 years or older. The most commonly reported barriers were the lack of spontaneous communication by patients regarding their sexual problems (82.1%), insufficient consultation time (60.7%), and barriers based on language/culture/religion (53.6%). The majority of the respondents (61.9%) expressed the need for training on discussing sexuality as a measure that may enhance the discussion of sexual life with patients. Most of the respondents (92.9%) considered the patients responsible for bringing up problems in their sexual functioning during a patient interview.

**Conclusion:**

Sexual dysfunction is rarely discussed with patients showing neurological diseases, particularly with female patients. This is due to the patient's inability to articulate their sexual problems freely as well as a lack of consultation time. Training on discussing sexuality may enhance the discussion of sexual life with patients.

## Background

Neurological diseases frequently affect sexual activity, and the resultant sexual dysfunction may be considered the most distressing feature of the disease by many patients (1). Neurological diseases can affect patients' response to sexual stimuli, change arousal and libido, and interfere with engorgement of the genitalia. Some epileptic phenomena can present as spontaneous engorgement and orgasm. Neurological diseases can also interfere with the physical ability to initiate and complete intercourse (2).

A previous study reported that 51% of patients with neurological diseases experienced a change in sexual life and that more than half of these patients, especially men, were concerned about these changes (3). The prevalence of sexual dysfunction after head trauma ranged from 36 to 54%, which is more than double that reported in healthy individuals without head trauma (4). The frequency of sexual dysfunction after stroke was similar to that reported after traumatic brain injuries (5). More than half of the patients with Parkinson's disease had sexual dysfunction (6). Among patients with epileptic, sexual dysfunction could be an ictal or interictal manifestation; more commonly, it occurs as a result of exposure to antiepileptic drugs, particularly hepatic P450 enzyme inducer (2). Similarly, 40–60% and 69.8% of men and women with multiple sclerosis, respectively, experience sexual dysfunction (7, 8).

Sexual dysfunction (SD) has a distressing effect on the patient's wellbeing and quality of life (QoL) (3). The available therapeutic agents for sexual impairment in patients with neurological diseases are limited, including sildenafil, which is very effective in the treatment of erectile impairment (4). Regardless of these limited therapeutic choices, talking about sexuality with the patient is still important, especially considering the wide diversity of sexual impairments, their high prevalence, and their detrimental effects on the wellbeing of patients and their spouses. Although the majority of neurologists consider the evaluation of sexual impairments an essential part of the treatment of neurological diseases, most often they omit to discuss sexual activity with their patients. Possible obstacles to initiating such discussions include the old age of the patients, relatively short interview time, or the patient's unwillingness to discuss this issue spontaneously (5). However, to the best of our knowledge, only a limited number of studies have explored the attitudes of neurologists regarding discussions of sexual impairment in patients with neurological diseases. Therefore, the current study aimed to highlight the issues impending such discussions and identify changes that may promote discussions on overcoming these issues.

## Methodology

This cross-sectional study was conducted among neurologists working in Saudi Arabia. Questionnaires and invitation letters were sent *via* email to the registered neurologist. The invitation letter included a request for approval from the respondents for the publication of the findings. Formal ethical approval is not required, since the survey included neurologist responses and did not involve patient data.

This survey was based on questionnaires used in previous studies (6–8).

It included 30 multiple-choice questions, with some questions also including free text. Our primary focus was to determine the possibility of discussing sexual activities and function with patients showing neurological diseases and the potential obstacles faced by neurologists in this regard. Other topics, such as the participants' opinion regarding who is responsible for discussing the patient's sexual life, the role of healthcare organizations, and the possibility of referral to other specialties; participants' knowledge about sexual dysfunction; and the importance of implementing this topic in training programs were also covered by the questionnaire. The participants were allowed to choose more than one response for some questionnaire items. Questions about participants' demographic data were included in the questionnaire, and the participants were offered an option to reject participation and asked for the possible cause of refusal.

### Statistical analyses

Statistical analyses were performed using the Statistical Package of Social Sciences (SPSS) program version 25, and a *p-*value of < 0.05 was considered to indicate statistical significance. Descriptive statistics were used for the demographic variables and responses to the questionnaire. Associations between categorical data were calculated by Pearson's chi-square test or Fisher's exact test. Numerical data were described as mean (standard deviation).

## Results

In the present study, 258 out of 750 neurologists (34.4%) returned the survey, of which 252 had completed the entire survey; therefore, their responses were considered suitable for further analysis.

The majority of the respondents were men (79.8%). The mean age of the participants was 36.1 (±5.9) years. Fifty-six percent of the respondent worked in a tertiary center, while 26.2% worked in a general hospital. The mean experience of practicing neurology among the participants was 10.3 (±5.7) years. Consultants of neurology represented 61.9% of the participants, while 15.5, 10.7, and 11.9% were senior registrar, registrar, and resident of neurology, respectively. The demographics of the participants were comparable with the neurologists working in Saudi Arabia (Table 1).

**Table 1:** Demographic characteristics of participants.

**Gender (*****N** **=*** **252) No. %**		
Male	201	79.8
Female	51	20.2
Age in years, mean (±SD)	36.1	±5.9
Mean years of practice in neurology (±SD)	10.3	±5.7
**Clinical level No. %**		
Consultant	156	61.9
Senior registrar	39	15.5
Registrar	27	10.7
Resident	30	11.9
**Clinical setting No. %**		
Tertiary or university hospital	141	56
General hospital	66	26.2
Private hospital	27	10.7
Private clinic	18	7.1

**Figure 1:**
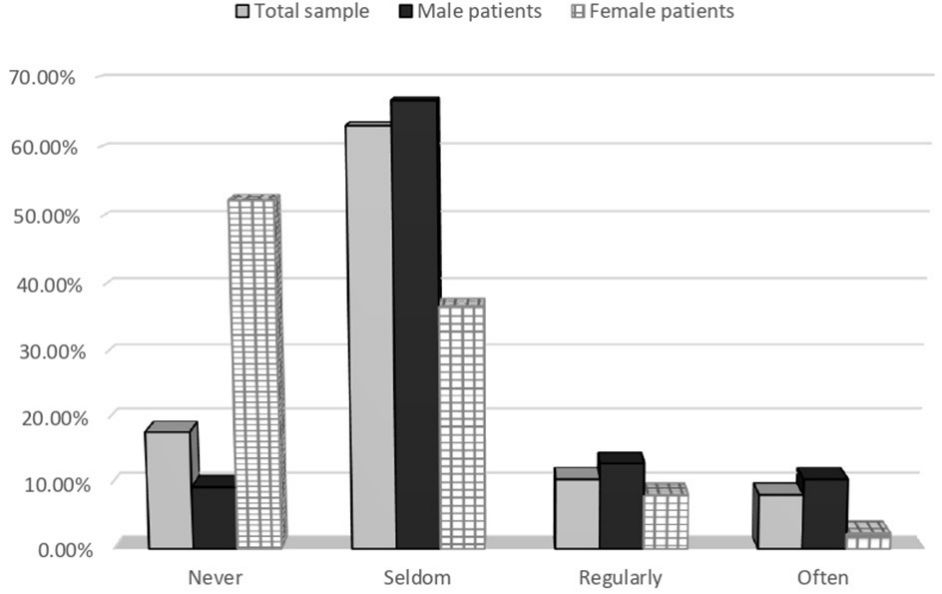
Discussing sexuality with patients with neurological disease, total results, and results in subgroups according to gender.

**Figure 2:**
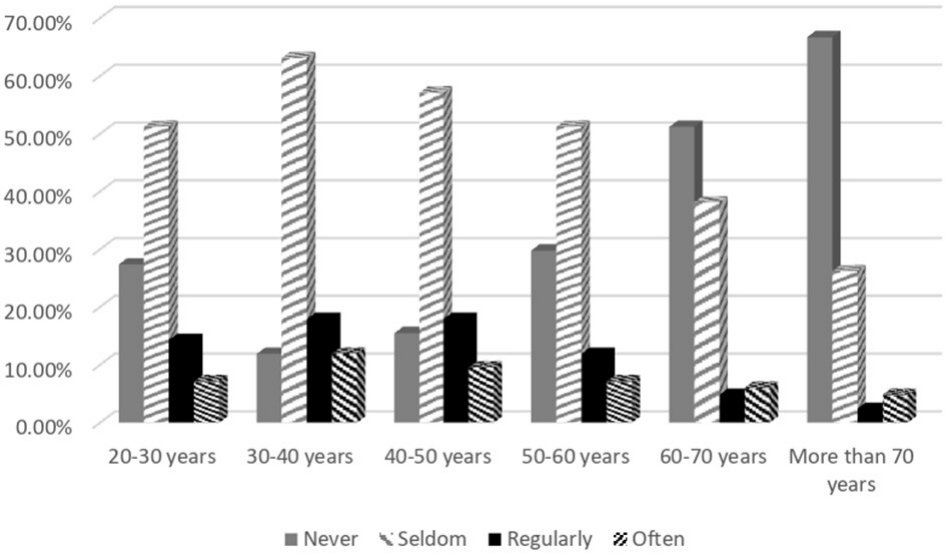
Discussing sexuality with patients with neurological disease, total results, and results in subgroups according to age group.

Table 2 categorizes the frequency of discussions of patient sexuality by neurologists. The majority of the respondents (63.1%) seldom discussed sexuality with their patients. Patients' sex and age influenced the responses; 52.4% never discussed this issue with female patients, however, 9.5% never discussed sexuality with male patients. More than half of the neurologists never addressed sexuality in patients aged 60 years or older, while more than 50% seldom addressed this issue with other age groups (Figures 1, 2).

**Table 2:** Discussing sexuality with patients with neurological disease, total results, and results in subgroups according to gender and age.

	**Never**		**Seldom**		**Regularly**		**Often**	
	**No**.	**%**	**No**.	**%**	**No**.	**%**	**No**.	**%**
Total sample	54	17.9	159	63.1	27	10.7	21	8.3
Male patients	24	9.5	168	66.7	33	13.1	27	10.7
Female patients	132	52.4	93	36.9	21	8.3	6	2.4
**Age groups**								
20–30 years	69	27.4	129	51.2	36	14.3	18	7.1
30–40 years	30	11.9	159	63.1	45	17.9	30	11.9
40–50 years	39	15.5	144	57.1	45	17.9	24	9.5
50–60 years	75	29.8	129	51.2	30	11.9	18	7.1
60–70 years	129	51.2	96	38.1	12	4.8	15	6
More than 70 years	168	66.7	66	26.2	6	2.4	12	4.8

**Figure 3:**
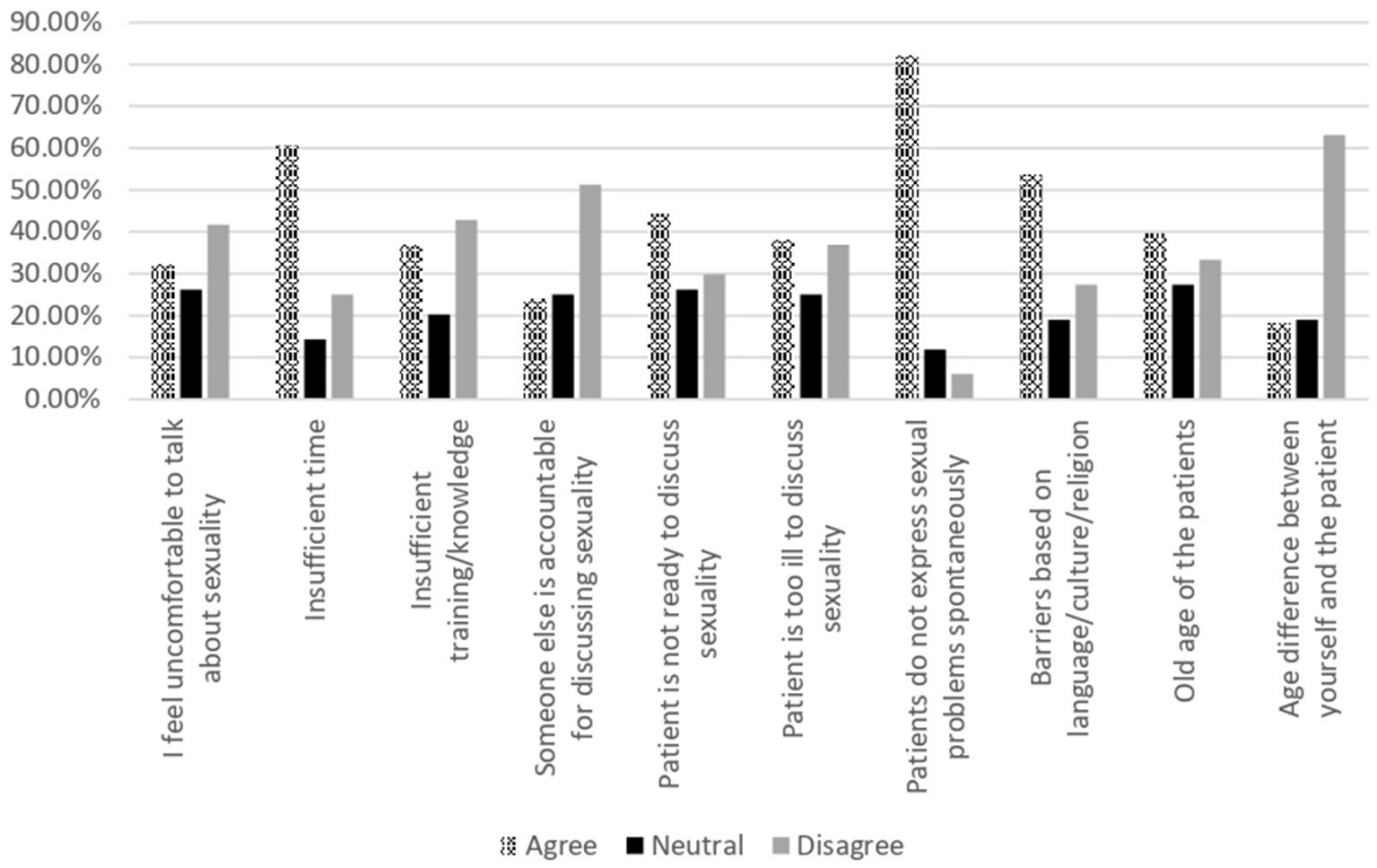
Barriers toward discussing sexuality.

The barriers that neurologists experienced in discussing patient sexuality are listed in Table 3 and Figure 3. The most commonly reported barriers were the lack of spontaneous communication by patients regarding their sexual problems (82.1%), insufficient consultation time (60.7%), and barriers based on language/culture/religion (53.6%). The majority of participants (63.1%) did not consider the age difference between the neurologists and patients as a possible barrier.

**Table 3:** Barriers toward discussing sexuality.

	**Agree**		**Neutral**		**Disagree**	
	**No**.	**%**	**No**.	**%**	**No**.	**%**
I feel uncomfortable to talk about sexuality	81	32.1	66	26.2	105	41.7
Insufficient time	153	60.7	36	14.3	63	25.0
Insufficient training/knowledge	93	36.9	51	20.2	108	42.9
Someone else is accountable for discussing sexuality	60	23.8	63	25.0	129	51.2
Patient is not ready to discuss sexuality	111	44.0	66	26.2	75	29.8
Patient is too ill to discuss sexuality	96	38.1	63	25.0	93	36.9
Patients do not express sexual problems spontaneously	207	82.1	30	11.9	15	6.0
Barriers based on language/culture/religion	135	53.6	48	19.0	69	27.4
Old age of the patients	99	39.3	69	27.4	84	33.3
Age difference between yourself and the patient	45	17.9	48	19.0	159	63.1

The majority (60.7%) of the neurologists reported that patients expressed sexual problems spontaneously in less than half of the cases. More than 70% of the participants reported that the patients' partners almost never spontaneously reported sexual problems and 75% of the participants almost never invited the patient's partners when they discussed sexuality. During the year before the questionnaire distribution, only 2.5% of the participants had referred more than 75% of neurological patients with sexual dysfunction to another care provider for counseling their sexual problems, while 51.9% referred less than a quarter of the patients. Most of the participants (78.6%) preferred to refer patients experiencing sexual problems to other care providers, especially urology clinics (Table 4).

**Table 4:** Participants' and patients' attitudes toward discussing sexual life.

	***N =* 252**	**%**
**How often do patients express sexual problems spontaneously?**		
Almost never	90	35.7
In half of the cases	3	1.2
In less than half of the cases	153	60.7
In more than half of the cases	6	2.4
**How often do patients' partners express sexual problems**		
**spontaneously?**		
Almost never	180	71.4
In half of the cases	12	4.8
In less than half of the cases	60	23.8
**How often do you invite the partner of the patient when you**		
**discuss sexuality?**		
Almost always	3	1.2
Almost never	189	75.0
In half of the cases	3	1.2
In less than half of the cases	48	19.0
In more than half of the cases	9	3.6
**What percentage of your patients suffering from chronic**		
**neurological disease did you refer to another care provider for**		
**counseling of their sexual problems over the last year?** ***N** =* **237**		
0	63	26.6
>25	123	51.9
25–50	33	14
51–75	12	5.1
76–100	6	2.5
**Is it possible to refer patients experiencing sexual problems to**		
**other care providers in your clinic?**		
No	24	9.5
Unknown	30	11.9
Yes	198	78.6
**To which specialty you will refer the patients? No**. = **186**		
Andrology	12	6.5
Psychiatry, urology, or gynecology	6	3.2
Urology	114	61.3
Urology or andrology	6	3.2
Urology or gynecology	30	16.1
Urology or psychiatry	15	8
Urology or Infertility specialty	3	1.6

The subject “sexual dysfunction in various neurological diseases” had not been implemented in the training program for neurology residents, according to 69% of the participants. Although more than half of the participants felt they were sufficiently competent to discuss sexuality with their patients and considered paying attention to sexual dysfunction in patients suffering from chronic neurological diseases as important, 44% had insufficient knowledge of sexual dysfunction and its treatment and 88.1% expressed the need to expand their knowledge to facilitate discussions on patient sexuality (Table 5).

**Table 5:** Participants' attitudes and opinions about knowledge in training in sexual life.

	***N =* 252**	**%**
**Is the subject “sexual dysfunction in various neurological**		
**diseases” implemented in the training program of neurology**		
**residents?**		
No	174	69.0
Yes	78	31.0
**How do you rate your own knowledge on sexual dysfunctions**		
**and the treatment of it?**		
A lot of knowledge	3	1.2
Insufficient knowledge	111	44.0
No knowledge at all	15	6.0
Some knowledge	87	34.5
Sufficient knowledge	36	14.3
**Do you feel competent to discuss sexuality to the patients?**		
No	123	48.8
Yes	129	51.2
**Are you in need of extending your knowledge on discussing**		
**sexuality?**		
No	30	11.9
Yes	222	88.1
**How important is it to pay attention to sexual dysfunction in**		
**patient suffering from chronic neurological disease?**		
Important	129	51.2
Indecisive	3	1.2
Slightly important	27	10.7
Unimportant	3	1.2
Very important	90	35.7

**Figure 4:**
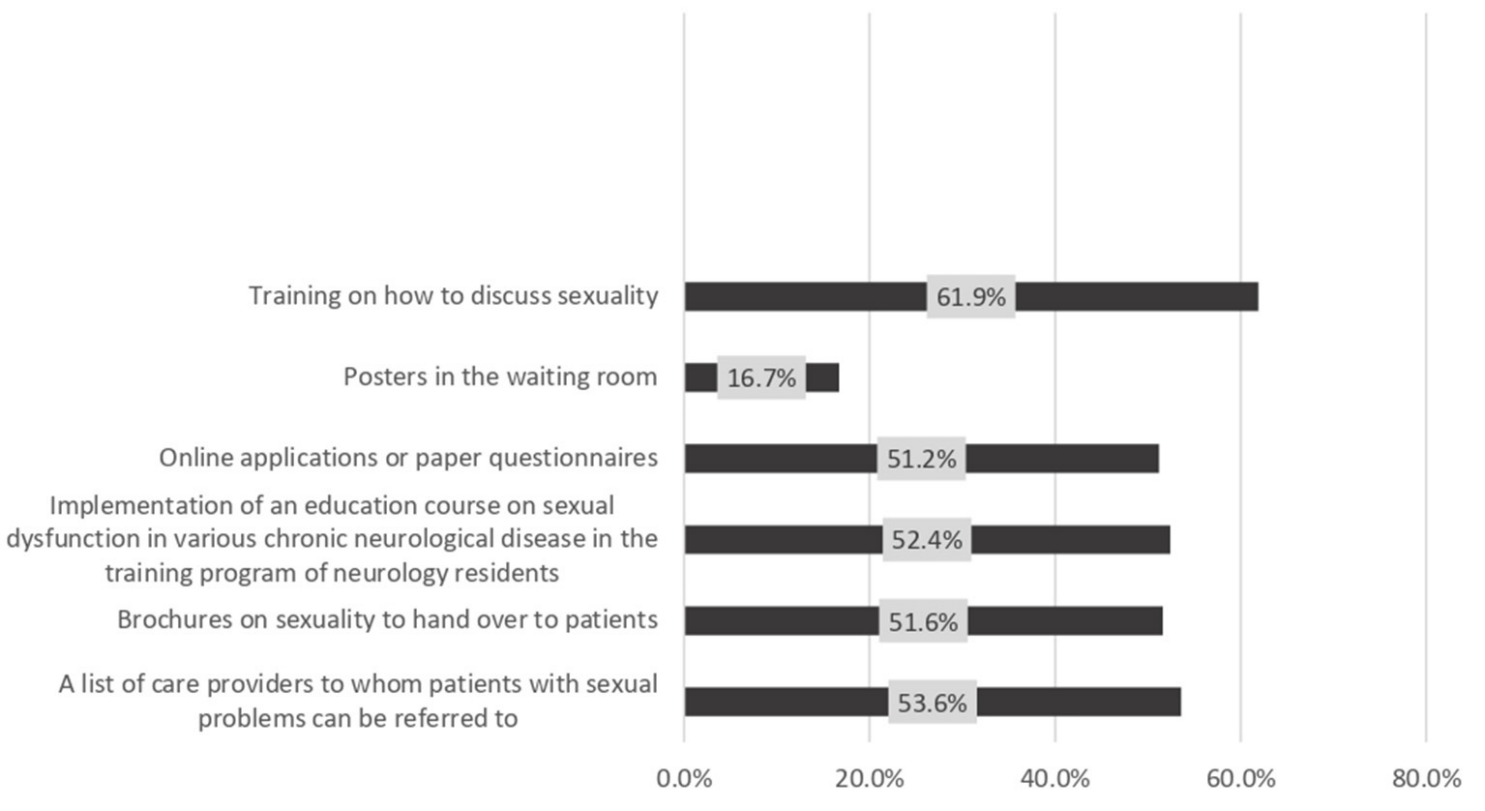
Facilities that might enhance the discussion on sexuality.

**Figure 5:**
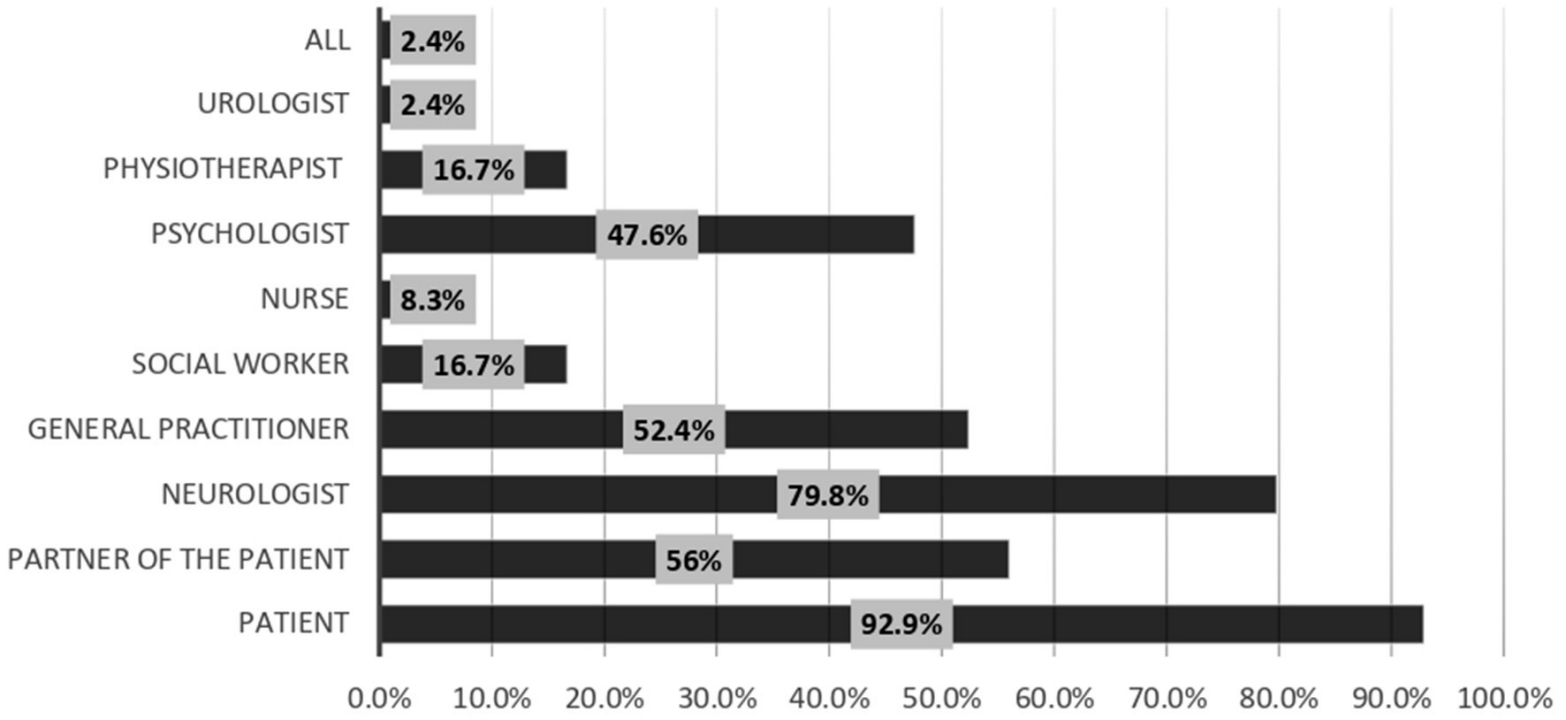
According to you, who is responsible for discussing sexuality?

**Figure 6:**
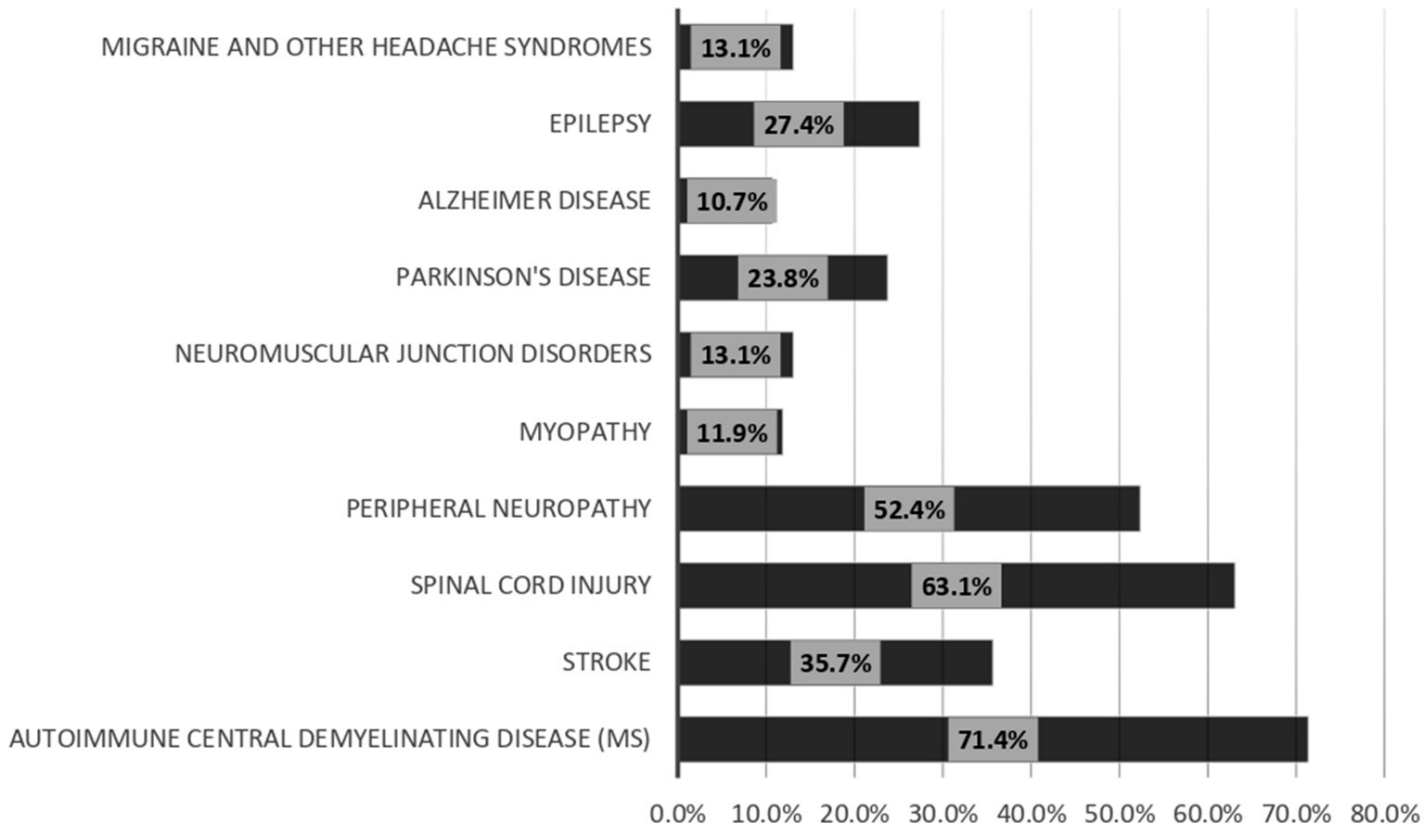
What type of disease you always discussing the sexual problem with it?

Regarding the measures that may enhance the discussion of sexual life with patients, 61.9% of the responders selected training on discussing sexuality, while 53.6% selected the availability of a list of care providers to whom patients with sexual problems can be referred. The least effective facilities were posters in the waiting room (16.7%) (Figure 4). Most of the respondents (92.9%) considered the patients responsible for bringing up problems in their sexual functioning, while 79.8% considered the neurologists responsible for discussing sexual function during a patient interview (Figure 5). Among the responders, 71.4 and 63.1% always discussed sexual dysfunction in patients with demyelinating disease and spinal cord lesions, respectively (Figure 6).

## Discussion

Neurological diseases have been recognized to be associated with a high prevalence of sexual dysfunction (3). We administered a questionnaire to evaluate the practice patterns of neurologists working in Saudi Arabia with regard to discussing sexuality with their patients. The majority of the respondents reported that they never or seldom discussed sexuality, especially with women and patients older than 50 years. To the best of our knowledge, this is the first study to examine the level of attention neurologists working in Saudi Arabia paid to the sexual function of their patients. The lack of discussion of sexuality especially with females could be due to cultural and religious reasons. Religion, culture, and society are influencing factors in forming our sexual thoughts, views, and expectations about sexuality. In middle east, due to its cultural context (conservative culture), speaking about sexual feelings and experiences for women is an undesirable practice, specifically for disable women. For this reason, less attention is paid to sexuality issues of women with neurological disease. Saudi Arabia has a conservative culture accordingly, it is not easy to discuss sexual problems with other gender, similar difficulties were reported in Iran (9), and cultural barriers have been reported as a major challenge to proper sexual discussion, and functioning (10). However, our results were in accordance with some previous studies conducted in different countries, and the study by van Hees et al. conducted among Dutch neurologists treating patients with Parkinson's disease (PD) reported that neurologists often omit to discuss sexuality with PD patients, especially with women and patients over the age of 70 years (11). A similar result was also reported in a multinational survey among neurologists conducted by Rooy et al. (12). Another study conducted among patients with multiple sclerosis also demonstrated under-evaluation of sexual function by the treating neurologists (13). The under-evaluation of female patients has been reported in other countries and diseases (14, 15).

Participants were asked about possible barriers that could interfere with the discussion of patient sexuality. According to their responses, the most common barrier was patients' unwillingness to express sexual problems spontaneously, followed by insufficient consultation time and barriers based on language, culture, and religion. This was consistent with the results reported by de Rooy et al., who noted that patients' inability to flag their sexual dysfunction was the largest barrier, especially for Spanish neurologists (12). Another study reported high patient age as the most frequently reported barrier (11); however, the authors had chosen neurologists specialized in PD. We think that this issue is an important problem since the majority of the participants reported that patients did not express sexual problems spontaneously, and patients' partners almost never do so either. Most of the participants reported that the neurologist should be responsible for discussing sexuality with the patients, and almost half considered it important to pay attention to sexual dysfunction in patients with chronic neurological diseases. In this situation, both patients and neurologists are hesitant to initiate discussions regarding sexual problems, which could be due to the insufficient knowledge that neurologists have about sexual dysfunction and its treatment in various neurological diseases. As reported by a considerable number of the participants, the training program for neurology residents does not include the topic of sexual dysfunction in various neurological diseases. Fortunately, the majority of the participants expressed the need to expand their knowledge of sexual dysfunction in various neurological diseases. The inclusion of knowledge regarding sexual dysfunction in training programs and the expansion of the number of courses on sexual dysfunction should resolve this issue.

Another problem highlighted by the study's findings was that few neurologists frequently referred neurological patients with sexual dysfunction to other care providers, especially urology clinics. Another suggested measure to improve the discussion of sexuality was the availability of a list of care providers to whom patients with sexual dysfunction could be referred. Nearly the same results were reported in the study by de Rooy et al., where most of the participants felt that it was necessary to increase their knowledge of discussing sexual problems and also reported that the participants would like brochures on sexual dysfunction to hand over to their patients (12). A previous study reported that the provision of information, education, and training to patients and physicians improved their motivation and confidence in discussing sexual problems (16–18). This is consistent with our results, wherein participants not only reported a need to increase their knowledge of sexuality but also preferred more materials to improve patients' information about sexual dysfunction-related issues.

The issue of sexual dysfunction was always discussed in patients with demyelinating disease and spinal cord lesions by most of the participants. Demyelinating diseases, especially multiple sclerosis, commonly occur between the ages of 20 and 40 years, when patients are normally sexually active (8). The majority of the respondents reported that they never or seldom discussed sexuality with patients older than 50 years, and most of them discussed sexuality at least regularly with younger age groups. The attitudes of the participants toward discussing sexuality with the younger age group could be the reason for the increased frequency of discussing sexuality with patients with demyelinating diseases. Spinal cord lesions are commonly associated with sexual dysfunction in men and women (19–22). Most neurologists are probably aware of the increased frequency of sexual dysfunction in patients with spinal cord injuries, which could explain the increased frequency of sexuality-related discussions in patients with spinal cord lesions.

Our study had some limitations. These included the dependence on self-report data, which may have resulted in an overestimation of answers, and the low response rate, which may indicate a lack of interest in this topic among neurologists. The present questionnaire does not cover respondents' perception regarding utility of asking questions about sex in neurological patients. We think this point should be explored in further research.

## Conclusion

The subject of sexual dysfunction is infrequently discussed while treating patients with neurological diseases, particularly female patients. The major reasons for the lack of such discussions are the inability of patients to express their sexual problems spontaneously and insufficient consultation time. This problem can be solved by improving patient awareness of sexual dysfunction, identifying a list of care providers to whom these patients can be referred, providing relevant training to neurologists, and improving their knowledge of discussing sexual problems. One of the limitations of the present study is that the present questionnaire does not include an item about respondents' perceptions regarding the utility of asking questions about sex in neurological patients. We suggest this point should be explored in further research. We recommend the inclusion of content regarding sexual dysfunction within neurologist training programs and expanding the number of courses on sexual dysfunction in various neurological diseases. Similar research done from a patient's perspective would add to the body of literature.

## Data availability statement

The original contributions presented in the study are included in the article/supplementary material, further inquiries can be directed to the corresponding author.

## Ethics statement

Ethics approval was granted by the Prince Sattam Bin Abdulaziz University Standing Committee of Bioethics Research (SCBR-041-2022). The patients/participants provided their written informed consent to participate in this study.

## Author contributions

DA reviewed the manuscript and pointed out the problems of the study. MK participated in data analysis and reviewed the drafts. DA, MK, and AA reviewed the manuscript. DA and MK presented the direction to the conclusion and finally checked the manuscript. All authors read and approved the final manuscript.
